# Gut microbiota, immunity, and bile acid metabolism: decoding metabolic disease interactions

**DOI:** 10.1093/lifemeta/load032

**Published:** 2023-07-23

**Authors:** Qixiang Zhao, Jiayu Wu, Yong Ding, Yanli Pang, Changtao Jiang

**Affiliations:** Center of Basic Medical Research, Institute of Medical Innovation and Research, Peking University Third Hospital, Beijing 100191, China; Department of Physiology and Pathophysiology, School of Basic Medical Sciences, State Key Laboratory of Vascular Homeostasis and Remodeling, Peking University, Beijing 100191, China; Center for Obesity and Metabolic Disease Research, School of Basic Medical Sciences, Peking University, Beijing 100191, China; Center of Basic Medical Research, Institute of Medical Innovation and Research, Peking University Third Hospital, Beijing 100191, China; Department of Physiology and Pathophysiology, School of Basic Medical Sciences, State Key Laboratory of Vascular Homeostasis and Remodeling, Peking University, Beijing 100191, China; Center for Obesity and Metabolic Disease Research, School of Basic Medical Sciences, Peking University, Beijing 100191, China; Center of Basic Medical Research, Institute of Medical Innovation and Research, Peking University Third Hospital, Beijing 100191, China; Department of Physiology and Pathophysiology, School of Basic Medical Sciences, State Key Laboratory of Vascular Homeostasis and Remodeling, Peking University, Beijing 100191, China; Center for Obesity and Metabolic Disease Research, School of Basic Medical Sciences, Peking University, Beijing 100191, China; Center of Basic Medical Research, Institute of Medical Innovation and Research, Peking University Third Hospital, Beijing 100191, China; Center of Basic Medical Research, Institute of Medical Innovation and Research, Peking University Third Hospital, Beijing 100191, China; Department of Physiology and Pathophysiology, School of Basic Medical Sciences, State Key Laboratory of Vascular Homeostasis and Remodeling, Peking University, Beijing 100191, China; Center for Obesity and Metabolic Disease Research, School of Basic Medical Sciences, Peking University, Beijing 100191, China

**Keywords:** intestinal microbiota, metabolic disease, immunity, bile acids

## Abstract

In recent decades, the global prevalence of metabolic syndrome has surged, posing a significant public health challenge. Metabolic disorders, encompassing diabetes, obesity, nonalcoholic fatty liver disease, and polycystic ovarian syndrome, have been linked to alterations in the gut microbiota. Nonetheless, the connection between gut microbiota and host metabolic diseases warrants further investigation. In this review, we delve into the associations between various metabolic disorders and the gut microbiota, focusing on immune responses and bile acid (BA) metabolism. Notably, T helper cells, innate lymphoid cells, macrophages, and dendritic cells have been shown to modulate host metabolism through interactions with intestinal microorganisms and the release of cytokines. Furthermore, secondary BA metabolites, derived from the microbiota, are involved in the pathogenesis of metabolic diseases via the farnesoid X receptor and Takeda G protein-coupled receptor 5. By covering both aspects of this immune system-microorganism axis, we present a comprehensive overview of the roles played by the gut microbiota, microbiota-derived BA metabolites, and immune responses in metabolic diseases, as well as the interplay between these systems.

## Introduction

Over the past few decades, with the improvement of living conditions and free access to high-caloric diets, a striking increase in the number of people with metabolic syndrome (MetS) worldwide has occurred, which has placed a great burden on human health and the global healthcare system [1]. The gut microbiota plays a critical role in host immune training, digestion of food, regulation of intestinal endocrine function and nerve signaling, alteration of drug action and metabolism, elimination of toxins, and the production of many compounds that affect the host [2]. Although common metabolic diseases, including type 2 diabetes (T2D) [3], obesity [4], non-alcoholic fatty liver disease (NAFLD) [5], and polycystic ovarian syndrome (PCOS) [6], differ greatly in pathology, they are associated with common and disease-­specific abnormalities in the composition and function of the gut microbiota.

Bile acid (BA)-modifying enzymes expressed by intestinal microbiota, including bile salt hydrolases (BSHs), metabolize primary BAs secreted by the liver and stored in the gallbladder into various secondary BAs [7]. Hydrophobic BAs at high concentrations reshape the gut microbiota composition mainly through membrane damage [8]. In addition to regulating the gut microbiota, these primary and secondary BAs influence the pathogenesis of metabolic diseases including NAFLD, T2D, and obesity by regulating receptors, including farnesoid X receptor (FXR) and Takeda G protein-coupled receptor 5 (TGR5) [9–12].

Chronic low-grade inflammation is a hallmark of metabolic diseases, suggesting that immune regulation, including adaptive immunity and innate immunity, plays an important role in affecting the course of metabolic diseases. Host immunity regulated by gut microbes has been reported to influence a variety of metabolic diseases. *Bacteroides vulgatus* aggravates PCOS by modulating BA profile and reducing type 3 innate lymphoid cell (ILC3)-produced interleukin (IL)-22 level [6]. Gut microbes, including segmented filamentous bacteria (SFB), prevent the development of obesity, MetS, and prediabetic phenotypes by inducing symbiotic-specific T helper 17 (Th17) cells [13].

In this review, we start with a description of the relationship between metabolic diseases and intestinal microbiota, and then highlight how this alters host immunity and BA metabolism. We then conclude with an overview of the crosstalk among BA metabolism, host immunity, and intestinal microbiota, as well as discussing the mechanisms by which these interactions involve intestinal microorganisms in various metabolic diseases.

## Intestinal microbiota and metabolic diseases

**Figure 1:**
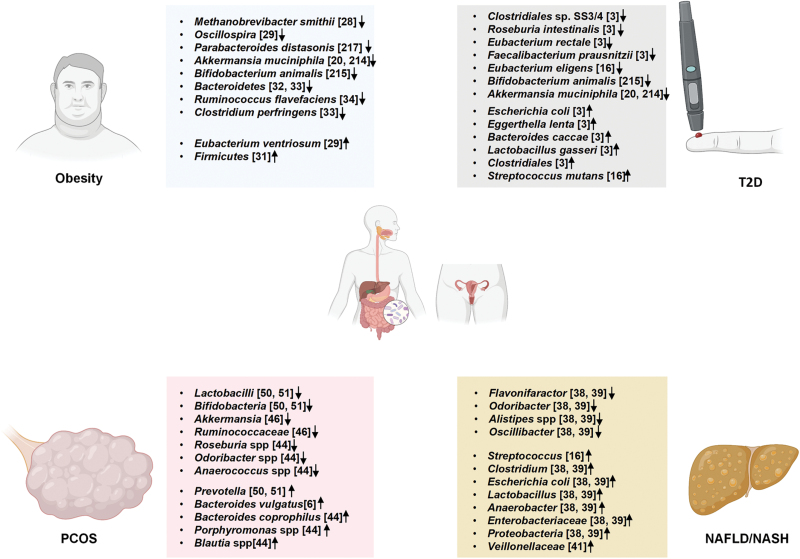
Intestinal microbiota features in metabolic diseases.

Observations over the past two decades have suggested that the intestinal microbiome may contribute in important ways to the metabolic health of the human host. Hence, we summarize the changes in the composition of gut microbes in metabolic diseases and their role in these diseases (Fig. 1 and Table 1).

**Table 1:** Intestinal microbiota in metabolic diseases.

Metabolic diseases	Intestinal microbiota	Change in abundance	Function
Obesity and T2D	*Escherichia coli*	↑	Unclear [3]
	*Clostridium*	↑	
	*Eggerthella lenta*	↑	
	*Bacteroides caccae*	↑	
	*Clostridiales* sp. SS3/4	↓	
	*Roseburia intestinalis*	↓	
	*Eubacterium rectale*	↓	
	*Faecalibacterium prausnitzii*	↓	
	*Lactobacillus gasseri*	↑	Unclear [16]
	*Clostridiales*	↑	
	*Lactobacillus*	↑	
	*Streptococcus mutans*	↑	
	*Eubacterium eligens*	↓	
	*Bacteroides intestinalis*	↓	
	*Roseburia*	↓	
	*Akkermansia muciniphila*	↓ [20, 244]	Improved metabolism in obese and diabetic mice [22]. Improved insulin sensitivity and decreased body weight in obese humans [21]
	*Bifidobacterium animalis*	↓ [245]	Attenuated obesity comorbidities in mice [246]
	*Parabacteroides distasonis*	↓ [247]	Alleviated mice obesity and metabolic dysfunctions [248]
	*Methanobrevibacter smithii*	↓	Unclear [32]
	*Oscillospira* spp.	↓	SCFAs production [249, 250]
NASH/NAFLD	*Streptococcus*	↑	Unclear [42, 43]
	*Clostridium*	↑	
	*Escherichia*	↑	
	*Lactobacillus*	↑	
	*Anaerobacter*	↑	
	*Flavonifaractor*	↓	
	*Odoribacter*	↓	
	*Alistipes* spp.	↓	
	*Oscillibacter*	↓	
	*Enterobacteriaceae*	↑	
	*Proteobacteria*	↑	
	*Escherichia* spp.	↑	
	*Veillonellaceae*	↑	Enhanced fecal BAs and propionate and instigated mice with the features of NAFLD [47]
	*Ruminococcaceae*	↑	
	*E. coli*	↑	Ethanol producing [48]
PCOS	*B. vulgatus*	↑	Modulated BA profiles and aggravated PCOS [6]
	*Prevotella*	↑	Unclear [56, 57]
	*Lactobacilli*	↓	
	*Bifidobacteria*	↓	
	*Akkermansia*	↓	Unclear [52]
	*Ruminococcaceae*	↓	
	*Bacteroides coprophilus*	↑	Unclear [50]
	*Porphyromonas* spp.	↑	
	*Blautia* spp.	↑	
	*Roseburia* spp.	↓	
	*Odoribacter* spp.	↓	
	*Ruminococcus bromii*	↓	
	*Anaerococcus* spp.	↓	

### T2D

Increasing evidence has shown the difference in intestinal microbiota composition and metabolic characteristics between patients with T2D and healthy individuals, as well as the relationship between the intestinal microbiota and whole-body metabolism [14, 15]. Two metagenome analyses in China and Europe have demonstrated the structural characteristics of the intestinal microbiota in patients with T2D and healthy individuals. These studies showed that the conditioned pathogens, such as some *Clostridium* species, *Escherichia coli*, *Eggerthella lenta*, and *Bacteroides caccae*, were increased, while butyrate-producing bacteria, including *Clostridiales* sp. SS3/4*, Roseburia intestinalis*, *Eubacterium rectale,* and *Faecalibacterium prausnitzii*, were decreased in Chinese patients with T2D [3]. In European patients with T2D, the abundance of *Lactobacillus gasseri*, some *Clostridiales* species, *Lactobacillus,* and *Streptococcus mutans* was increased. Like the Chinese patients with T2D, a decrease in the abundance of butyrate-producing bacteria, including *Eubacterium eligens*, *Bacteroides intestinalis*, and *Roseburia*, was found [16]. The abundance of *Akkermansia muciniphila*, an emerging potential probiotic, was inversely correlated with the presence of overweight and diabetes in human and murine studies [17–19]. In addition, oral administration of *A. muciniphila* improved insulin resistance (IR) in diabetic mice, while decreasing body weight and the levels of blood markers related to liver dysfunction in humans with overweight and obesity [20, 21]. However, it is important to note that the clinical trial mentioned has limitations due to its small sample size, with a total of only 32 participants divided into three groups [21]. As a result, drawing definitive conclusions from such a study can be challenging. It is worth mentioning that a significant portion of the existing researches on *A. muciniphila* and its potential effects on obesity and T2D are derived from animal or preclinical studies [20, 22, 23]. While these studies provide valuable insights, the translation of findings from animal models to human subjects may not always be straightforward. To establish a more comprehensive understanding of the potential ameliorative effects of *A. muciniphila* on obesity and diabetes, further large-scale clinical trials with robust methodologies are necessary. These trials will provide more substantial evidence and help determine the true therapeutic potential of *A. muciniphila* in the context of obesity and diabetes. Like *A. muciniphila*, another short-chain fatty acids (SCFAs)-producing bacterium, *Bifidobacterium animalis*, has also been reported to be negatively associated with T2D [24].

### Obesity

A poor lifestyle and dietary habits increase the probability of obesity, while evidence has shown that widespread use of antibiotics aggravates obesity, suggesting an important role of the intestinal microbiota in maintaining a proper body weight [25, 26]. Alyssa *et al*. concluded that antibiotic use can disrupt the gut microbiota, leading to changes in energy metabolism, increased adiposity, and obesity [27]. Nonetheless, a clinical study showed that the use of narrow-spectrum antibiotics (including penicillin and amoxicillin) was not associated with the development of obesity in infants while the use of broad-spectrum antibiotics was associated with early childhood obesity [28]. These studies suggest that not all intestinal microbes are related to obesity. Earlier studies have shown that a transferrable obesity-associated microbiota induces weight gain in lean mice [29]. Furthermore, twin studies have demonstrated that the abundance of bacterial producers of SCFAs, such as *Eubacterium ventriosum*, is associated with obesity, whereas butyrate producers, such as the methanogenic archaeon *Methanobrevibacter smithii* and *Oscillospira* spp., may be associated with leanness [30–33]. Meanwhile, other evidence has shown that SCFAs and SCFA producers contribute to the amelioration of obesity [34]. Administration of *A. muciniphila* and *B. animalis*, two SCFA-producing bacteria, improves metabolic indices in patients with obesity [21, 24]. Moreover, several studies have focused on dynamically linking changes in the levels of the major bacteria *Bacteroides* and *Firmicutes* to obesity and weight loss [35]. Compared with lean individuals, individuals with obesity presented a reduced proportion of *Bacteroidetes* and higher levels of *Firmicutes*. Interestingly, the relative abundance of *Bacteroidetes* increased while that of *Firmicutes* decreased after a dietary intervention to treat obesity [36]. Zuo *et al*. showed that the amount of *Bacteroidetes* and *Clostridium perfringens* was significantly lower in patients with obesity than in normal-weight individuals [37]. However, another study reported that the median proportion of *Bacteroidetes* was higher in individuals with overweight and obesity than lean participants, while *Ruminococcus flavefaciens*, a bacterial division of *Firmicutes*, was lower in patients with overweight and obesity, indicating that analysis for intestinal microbiota should be refined to the species classification [38].

### NAFLD/non-alcoholic steatohepatitis (NASH)

NAFLD, which encompasses various forms of liver pathology, from simple hepatic steatosis to NASH, the inflammatory and aggressive form of NAFLD [39], has been frequently regarded as the hepatic manifestation of MetS. In many countries, the incidence of NAFLD or NASH is up to 20%–40% of adults, thus representing a very large unmet clinical need [40]. Increasing evidence has shown that the liver and gut are interdependent at multiple levels, and disturbance of the gut–liver axis has been implicated in a number of conditions linked to obesity, including NAFLD [41]. Patients with NAFLD have an increased abundance of *Streptococcus*, *Clostridium*, *Escherichia*, *Lactobacillus*, and *Anaerobacter*, while they have a decreased abundance of *Flavonifaractor*, *Odoribacter*, *Alistipes* spp., and *Oscillibacter* [42]. The abundance of *Enterobacteriaceae*, *Proteobacteria*, and *Escherichia* spp. is elevated in individuals with NASH [43]. To better understand the relationship between NASH/hepatic fibrosis and intestinal microbiota, researchers explored the intestinal microbiome as a biomarker to distinguish simple steatosis from NASH and NAFLD to cirrhosis. Based on a random forest machine learning algorithm, authors identified intestinal microbes that changed during different stages of NALFD, including the increased *Veillonella parvula* and decreased *E. eligens* during aggravation of fibrosis [44]. Besides, another study demonstrated that *Clostridium spiroforme*, *Eubacterium dolichum*, *Streptococcus anginosus*, and *Veillonella dispar* were significantly correlated with NAFLD progression, whereas *Annona senegalensis*, *Lactobacillus crispatus*, *Limosilactobacillus vaginalis*, and *Weissella cibaria* were significantly negatively associated with NAFLD progression (NAFLD, NASH, fibrosis, and cirrhosis) [45]. We previously showed that *Bacteroides xylanisolvens* degraded gut nicotine and alleviated smoking-related NASH [46]. In addition, the abundance of *Veillonellaceae* and *Ruminococcaceae* was correlated with fibrosis in lean individuals accompanied by enhanced fecal BAs and propionate, and administration of *Veillonellaceae* and *Ruminococcaceae* alleviated liver damage in NAFLD mouse models [47]. Given that the microbiota associated with NALFD is enriched in ethanol-producing bacteria, such as *E. coli*, it has been hypothesized that the aberrant gut microbiomes of individuals with NAFLD produce more ethanol than microbiomes of healthy individuals, as evidenced by increased concentrations of intrinsically generated ethanol in the circulation and breath [43, 48, 49].

### PCOS

The relationship between PCOS and changes in intestinal microbiota has been the subject of numerous studies in recent years, which have shown significant differences in gut microbiota composition between patients with PCOS and healthy controls [50–52]. In addition to an alteration in α and β diversity in the gut microbiome, the studies showed that there is also an alteration in the balance of some species of bacteria, including *Bacteroidetes* and *Firmicutes*, in patients with PCOS [53]. At the genus level, *Bacteroides*, *Escherichia*/*Shigella*, and *Lactobacillus* were higher in patients with PCOS [54]. Furthermore, the relative abundance of *Bacteroides coprophilus*, *Porphyromonas* spp., and *Blautia* spp. was consistently higher in patients with PCOS, while *Roseburia* spp., *Odoribacter* spp., *Anaerococcus* spp., and *Ruminococcus bromii* were significantly lower [50]. Our research demonstrated that the abundance of *B. vulgatus* was markedly greater in patients with PCOS compared with healthy controls, and administration of *B. vulgatus* aggravated PCOS in mice [6]. In addition, beneficial bacteria, such as *Lactobacilli* and *Bifidobacteria* which enhance immunity and nutrient absorption, were instead significantly lower in patients with PCOS, while level of *Prevotella*, a proinflammatory gut microorganism, was greater in patients with PCOS, which may induce an adverse inflammatory effect in the host [55–60].

## BA metabolism in metabolic diseases

Primary BAs are converted from cholesterol in the liver to taurine and glycine conjugates, which are secreted into the intestines, where they are transformed into secondary BAs [61]. These transformations form a BA pool with extensive diversity. Bacterial-transformed secondary BAs activate different BA receptors to regulate signaling pathways with broad coverage of complex symbiotic metabolism networks, including glucose metabolism, lipid metabolism, energy homeostasis, and inflammation [62]. These BA receptors include the liver X receptor, FXR, pregnane X receptor, and G protein-coupled receptors (GPCRs), such as TGR5. Here, we highlight the role of BAs in regulating metabolic diseases through these BA-binding receptors.

### FXR

The major regulator of BA homeostasis is FXR, a ligand-activated member of the nuclear receptor superfamily [63], which can be bound by a number of endogenous BAs, including taurocholic acid (TCA), lithocholic acid (LCA), deoxycholic acid (DCA), chenodeoxycholic acid (CDCA), cholic acid (CA), and muricholic acid (MCA), at various affinities [64, 65]. In a previous paper, we explored the role of the FXR-BA axis in regulating metabolic disease [11], and here we summarize the role of BAs in metabolic disease. Notably, early studies revealed that *Fxr*-null mice had elevated triglyceride and serum cholesterol levels, and increased liver mass and peripheral IR when fed a standard low-fat diet [66, 67]. However, these mice had reduced adipocyte size and were protected from high-fat diet (HFD)-induced obesity and genetically induced obesity, as well as associated IR, suggesting an important role of FXR signaling in obesity and diabetes [68]. In a BA-FXR-ceramide axis, hepatic BA synthesis and intestinal microbiota-mediated BA deconjugation were upregulated upon HFD feeding, which resulted in increased levels of the endogenous FXR agonists CA, CDCA, DCA, and LCA. Intestinal FXR activation-induced genes (*Smpd3/4*, *Sptlc2,* and *Cers4*) involved in ceramide synthesis, which contributes to increased circulating ceramide levels that in turn promote metabolic disorders [69]. Dietary and drug interventions can regulate the abundance of BSH-secreting gut microbiota and these strategies can modulate BA deconjugation and levels of the endogenous FXR antagonists glycoursodeoxycholic acid (GUDCA), tauroursodeoxycholic acid (TUDCA), and tauro-β-muricholic acid (TβMCA), which contributes to an improvement in metabolic diseases. One study showed that glycine-β-muricholic acid (Gly-MCA) ameliorates NASH in mice by inhibiting intestinal FXR and reducing the production of ceramides [70]. Several of our studies have also illustrated the role of an intestinal FXR-ceramide axis in NASH. Mice treated with tempol or antibiotics exhibited altered BA composition, including the increased abundance of TβMCA, leading to fewer circulating ceramides and reduction of hepatic triglyceride accumulation. The administration of ceramides attenuated the effects of antibiotic treatment on the development of HFD-induced, intestinal FXR-dependent NAFLD [10]. In addition to regulating the production of ceramides by FXR, BAs can also regulate FXR by the production of fibroblast growth factor 15 (FGF15, FGF19 in humans) and TGR5, thus regulating metabolic disorders [69]. Different from the promotional effects of ceramide toward metabolic disorders, activation of intestinal FXR with an intestine-restricted agonist, fexaramine, results in decreased HFD-induced metabolic phenotypes in mice by increasing FGF15 synthesis, which is delivered to the liver where it decreases the expression of the hepatic BA synthesis enzyme cytochrome P450 family 7 subfamily A member 1 (CYP7A1) [71]. Later work found the potential mechanism. Notably, fexaramine alters the intestinal microbiota composition, including increasing the levels of *Acetatifactor* and *Bacteroides*, which are the predominant bacteria that convert CDCA and ursodeoxycholic acid (UDCA) to LCA, a secondary BA that acts as a TGR5 agonist [72]. However, a study showed that treatment with fexaramine failed to raise the concentrations of TGR5 ligands, modulate TGR5 signaling, or improve dysmetabolic status [73].

FXR, as an important receptor regulating metabolism, has attracted significant attention in drug development. Several licensed drugs targeting FXR have been reported. Obeticholic acid (OCA), a semisynthetic analog of CDCA, has shown potency ~100-fold higher than CDCA. It inhibits BA synthesis and reduces BA levels in hepatocytes. OCA has been approved for the treatment of primary biliary cholangitis, demonstrating efficacy in improving liver function and reducing alkaline phosphatase levels [74, 75]. In addition, based on the beneficial effects on hepatic inflammation and also on glucose and lipid metabolism, OCA has been shown to improve the histological features of NASH in a Phase 3 trial, including fibrosis [76]. 24-nor-ursodeoxycholic acid (norUDCA) (renamed as norucholic acid) is also a therapeutic modified BA that has shown promising results in Phase II clinical trials for the treatment of primary sclerosing cholangitis (PSC) [77]. Zhu *et al.* demonstrated that norUDCA ameliorates intestinal inflammation by regulating the intestinal Th17/regulatory T (Treg) cells balance and restricting glutaminolysis during Th17 cell differentiation [78]. As an oral ileal BA transporter inhibitor, elobixibat improves symptoms in patients with functional constipation by increasing BA levels in the colon [79].

### TGR5

Gut microbiota-derived LCA activates TGR5 (which is expressed in enteroendocrine L cells) and induces the expression of the gene encoding TGR5, resulting in increased secretion of glucagon-like peptide 1 (GLP-1), thereby improving insulin sensitivity and reducing obesity through white adipose browning [72, 80, 81]. 6a-ethyl-23(S)-methyl-cholic acid (INT-777), a TGR5 agonist, increases energy expenditure and reduces hepatic steatosis and adiposity in diet-induced obesity (DIO) mice [82]. In addition, 6α-hydroxylated BAs, produced from primary BAs by intestinal bacteria when fed a Western-style diet, improve glucose metabolism via TGR5 signaling [83]. In a previous study, we identified hyocholic acid as an agonist of TGR5 and an inhibitor of FXR, improving glucose homeostasis [9]. In addition, we revealed a positive correlation between elevated TCA and DCA levels and activation of TGR5. This activation upregulated the expression of mitochondrial creatine kinase 2 and uncoupling protein 1, leading to an elevation of white adipose tissue thermogenesis [84]. Furthermore, glycodeoxycholic acid (GDCA) treatment improves IR, ovarian dysfunction, and infertility in mice with PCOS, while prevention of TGR5 signaling in Tgr5^−/−^ mice significantly reduced the enhanced IL-22 secretion by ILC3s [6]. An interesting study showed that genetic downregulation of hypothalamic TGR5 expression in the mediobasal hypothalamus promoted the development of obesity and aggravated established obesity by blunting sympathetic activity [85].

Furthermore, in addition to their binding to receptors such as FXR and TGR5, BAs have the capability to directly interact with mitochondria and regulate metabolic processes associated with the MetS. Specifically, DCA and taurodeoxycholic acid have been shown to induce mitochondrial reactive oxygen species (ROS) production and promote the activation of mitochondria-dependent receptor tyrosine kinases [86]. UDCA has demonstrated positive effects on glycemic parameters, insulin sensitivity, and surrogate markers of IR in patients with NASH [87, 88]. Researchers found that UDCA decreased lipid droplets (LDs), free fatty acids (FFAs), and triglycerides (TG) by improving mitochondrial function, including reduced ROS production and improved mitochondrial swelling [89]. Besides, CDCA was reported to suppress the progression of acute myeloid leukemia through synergistically promoting LD accumulation and lipid peroxidation via ROS/p38 mitogen-activated protein kinase/diacylglycerol O-acyltransferase 1 pathway, which is caused by mitochondrial dysfunction [90].

## Intestinal and peripheral immunity in metabolic diseases

**Figure 2:**
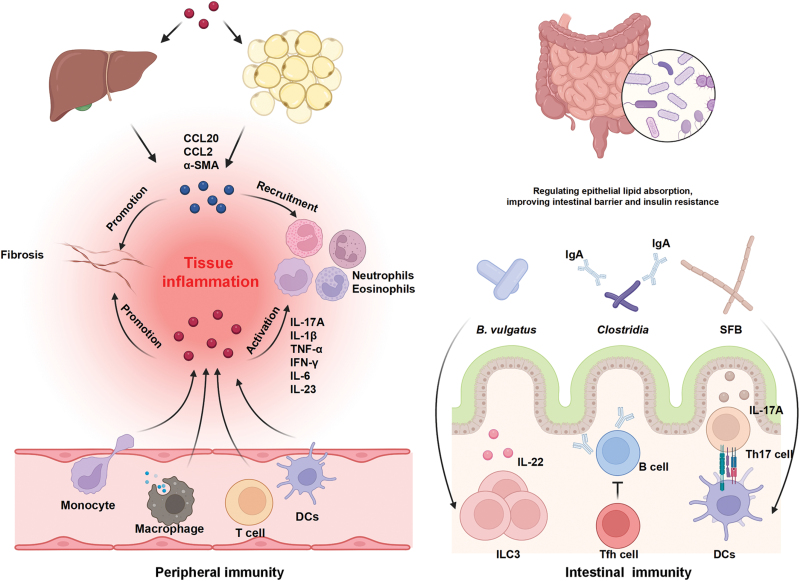
Intestinal and peripheral immunity in metabolic diseases.

Aberrations in the communication between the adaptive and innate immune systems and the intestinal microbiota might contribute to complex diseases [91, 92]. The important relationship between metabolic diseases and host immunity has been widely reported. Given that point, we summarize here the changes in immune cells and related cytokines that occur in metabolic diseases along with the contributions of their regulations toward metabolic diseases (Fig. 2).

## Peripheral immunity in metabolic diseases

### Macrophages

Among immune cells infiltrating obese adipose tissue, macrophages dominate both in function and number [93]. In the adipose tissue of mice, the ratio of F4/80^+^ cells in obese mice is significantly higher than that in lean mice [94]. In addition to the number, adipose tissue macrophages (ATMs) exhibit significant cellular localization and inflammatory potential [95]. In lean animals, ATMs predominately display an alternatively activated (M2) phenotype, including the secretion of IL-10, and are less inflammatory than classically activated macrophages and uniformly dispersed throughout the adipose tissue. ATMs have a proinflammatory, classical (M1) phenotype, as characterized by the secretion of proinflammatory cytokines, and are primarily found in “crown-like” structures around dying adipocytes in obese mice [96–99]. Regarding the mechanism by which macrophages regulate obesity, cyclooxygenase 2, the rate-limiting enzyme in the biosynthesis of prostaglandin E2 (PGE2), limits adipose tissue dysfunction in obese mice dependent on PGE2 receptor subtype, E-type prostanoid receptor 4 [100].

The contributions of macrophages to the pathogenesis of NASH have been characterized most extensively in the liver. Pathways driven by macrophages include not only fibrosis and inflammation but also steatosis via secretion of tumor necrosis factor alpha (TNF-α), IL-1β, CC-chemokine ligand 2, and monocyte chemoattractant protein-1 (MCP-1) [101]. During NASH, inflammatory signals drive the hepatic recruitment of blood monocytes, which differentiate into monocyte-derived macrophages, thereby increasing the size of the macrophage pool in the liver. Recent studies have shown the diversity of hepatic macrophages in mouse models of NASH. During NASH, embryonically derived Kupffer cells (KCs), a class of resident macrophages present in the sinusoidal sinuses of the healthy liver, are gradually lost. Instead, circulating Ly6C^+^ monocytes are recruited to the liver, where they differentiate into monocyte-derived KCs to maintain the size of the KC pool. Compared with embryonically derived KCs, monocyte-derived KCs are more proinflammatory and affect the liver response to NASH by limiting liver lipid storage and promoting liver injury [102]. With lipid-associated macrophages identified to regulate metabolic homeostasis in a myeloid cell-2 (TREM2)-dependent manner [103]. TREM2 was reported to maintain macrophage-hepatocyte metabolic coordination in NAFLD and prolonged hyper nutrition leads to the production of proinflammatory cytokines, which induces TREM2 shedding through A disintegrin and metallopeptidase domain 17 (ADAM17)-dependent proteolytic cleavage and thus exacerbates the progression of NASH [104, 105]. In our research, we demonstrated that macrophage hypoxia-inducible factor 2-alpha (HIF-2α) alleviates IR by inhibiting NLRP3 [nucleotide-binding domain leucine-rich repeat (NLR) and pyrin domain containing receptor 3] inflammasome activation in a carnitine palmitoyltransferase 1a (CPT1A)-mediated, fatty acid oxidation (FAO)-enhanced manner [106]. Furthermore, macrophage HIF-1α activation triggers vascular inflammation and aggravates aortic dissection by increasing the expression and activity of ADAM17 [107].

### T cells

Conventional T cells, expressing the αβT-cell receptor, represent more than 50% of the whole lymphocyte population. They recognize antigen peptides bound to their major histocompatibility complex protein on antigen-presenting cells and are mainly classified into CD8^+^ and CD4^+^ T cells. CD8^+^ cells are involved in macrophage activation, differentiation, and migration, thereby initiating inflammatory cascades in adipose tissue, thus leading to IR. Nevertheless, regarding adaptive immunity, CD4^+^ T cells seem to play a more important role in obesity and IR [108]. Interferon-γ (IFN-γ)–expressing CD4^+^ cells increase in obesity, and IFN-γ deletion improves obesity-induced IR and lowers macrophage infiltration in adipose tissue [109, 110]. A study demonstrated that DIO is predisposed to an increased Th17 bias [111]. Similarly, an increased number of Th17 cells and levels of IL-17 were also observed in adipose tissue and liver tissue in mice with DIO [112–114]. However, interesting findings were observed in the gut of obese mice fed an HFD, which demonstrated that the number of Th17 cells and levels of IL-17 were decreased [115, 116], suggesting different functions of Th17 cells in intestines, which will be discussed in the intestinal immune section below. A number of studies have shown that a dramatic reduction of visceral adipose tissue (VAT) Tregs occurs during DIO in mice [109, 117–119]. Like the findings in mice models, the number of CD4^+^Foxp3^+^ Treg is decreased in adipose tissue and peripheral blood from adult individuals with obesity and diabetes [120–122]. Rag1^−/−^ mice, lacking lymphocyte immune populations, showed a reversal of the NASH phenotype, suggesting the important role of lymphocytes in the pathogenesis of NASH [123]. Similarly, the depletion of CD8^+^ T cells also prevented liver damage and inflammation without altercating the systemic metabolic phenotype, providing clear evidence for the role of CD8^+^ T cells and their secreted cytokines in NASH development. These findings were reproduced in different nutritional and genetic NASH models associated with obesity; however, CD8^+^ cells seem not to play a central role in models of lean NASH [124, 125]. Th1 cells have been reported to be increased in individuals diagnosed with NASH and in obese mice fed high-caloric diet; however, their role in NASH has been poorly explored, and controversial evidence in experimental murine models exists [126, 127]. Like Th1, published data about Th2 cells and NASH are scarce. In mesenteric lymph nodes, the ratio of Th1/Th2 was shown to be altered in murine models of NAFLD [128]. In humans, the transition from simple fatty liver to NASH was shown to be determined by increased hepatic Th17 cells, expression of IL-17, and reduced frequency of Tregs [129]. IL-17 directly induces type I collagen production in hepatic stellate cells (HSCs) through the signal transducer and activator of transcription 3 pathway, and IL-17 receptor knockout inhibits the formation of hepatic fibrosis [130]. Single-cell metabolomics revealed that the regulation of IL-17A on the metabolic status of liver cells toward steatosis subsets depends on nuclear factor kappaB (NF-κB) [131], and a distinct pathogenic subgroup of liver inflammatory hepatic CXCR3^+^ Th17 (ihTh17) cells in NALFD mice and human patients was identified. Increased numbers of ihTh17 cells were observed in patients and mice with NALFD, and they accelerated the progression of NAFLD through a PKM2-dependent metabolic skewing [132]. In addition to αβT cells, a study focused on γδT cells showed that exogenous commensal lipid antigens augment the hepatic γδT-17 cell number and the microbiota accelerated NAFLD through hepatic γδT-17 cells [133]. At present, few studies on T cells and PCOS have been reported. According to a clinical study, researchers found that the percentage of Treg cells was significantly lower while the percentage of the Th17 cells of the PCOS group was significantly higher than that of the control group. In addition, the ratio of Treg/Th17 was significantly lower in the PCOS group [134].

### ILCs

The ILC family consists of cytotoxic cells (natural killer (NK) cell) and non-cytotoxic subsets (ILC1, ILC2, and ILC3). Here we mainly discuss the role of NK cells and ILC3s in metabolic diseases. In humans, several studies have shown that the NK cell number is reduced in the peripheral blood in patients with obesity [135, 136]. However, murine models of obesity demonstrated NK cell accumulation in adipose tissue [137, 138]. One explanation for this disparity is that adipocytes in obesity secrete more MCP-1 and infiltrating NK cells in adipose tissue express higher levels of MCP-1 receptors, such as NKG2D, CD158, and NKp46, which are recruited into the adipose tissue to promote inflammation [139–141]. It is generally agreed that the activation and proliferation of NK cells in the VAT in the context of obesity also play important roles in IR and T2D through interaction with ATMs, triggering and amplifying the secretion of inflammatory cytokines, such as TNF-α and IL-6, by macrophages [142, 143]. The activity and frequency of NK cells in the adipose tissue were comparable in healthy individuals and those with NAFLD or NASH [144]. However, studies in mice have shown an increased accumulation of NK cells in adipose tissue after feeding with an HFD that presents a transcriptional profile different from control diet-fed mice, including increased IL6Ra and the myeloid marker Csf1 [145]. Emerging evidence suggests that ILC3s may regulate obesity or metabolic homeostasis through the secretion of IL-17A or IL-22 [146]. CCR6^+^ ILC3-derived IL-17A promotes the pathogenesis of obesity-associated airway hyperreactivity in Rag1^−/−^ mice that are dependent on HFD-induced, macrophage-derived IL-1β [147]. IL-22 is required for the prevention of obesity and IR through the regulation of triglyceride lipolysis and FAO in adipocytes. Mice lacking the IL-22 receptor are more susceptible to HFD-induced obesity and IR, and treatment of obese mice with IL-22 suppresses TNF expression in adipose tissue, as well as improving IR [148]. However, ILC3-derived IL-22 can also contribute to metabolic disease [149, 150]. A recent paper reconciled these seemingly contradicting reports and suggested that the role of ILC3s is context dependent [13]. The authors pointed out that ILC3s provide protection from metabolic disease in the absence of Th17 cells and when Th17 cells and Th17-inducing gut microbes are present, ILC3s counteract the protective effects of Th17 cells and promote the pathogenic effects of an HFD [13]. Similar to the protective effect of ILC3s in obesity, ILC3-derived IL-22 protects against obesity-associated NAFLD through the improvement of hepatic lipid metabolism and inhibition of palmitate-induced primary hepatocyte apoptosis [151]. Some published studies have shown a decreased level of RORγt^+^ IL-22^+^ ILC3s in blood and intestinal samples of patients with PCOS and animal models [51].

### Dendritic cells (DCs)

Dendritic cells are part of a large and complex group of cells that comprise two major classes, notably, plasmacytoid DCs (pDCs) and conventional or classical DCs (cDCs) [152]. Upon local activation, DCs migrate to tissue-draining lymph nodes (DLNs) where they interact with naive T cells [153]. The presence of cDCs in the VAT has been reported in numerous studies. In patients, the presence of CD11c^+^ CD1c^+^ DCs correlates with the body-mass index (BMI) and an elevation in Th17 cells [154]. In addition, the depletion of CD11c^+^ cells (including CD11c^+^ monocytes/macrophages) results in the normalization of insulin sensitivity and the decrease of proinflammatory cytokines in obese mice [155, 156]. However, mice lacking conventional type 1 DCs (cDC1s) gained weight and were obese during aging, and another study showed that cDC1s promote an anti-inflammatory environment in the body that delays the development of obesity-associated chronic inflammation and IR via an IL-10-dependent activation of the Wnt/β-catenin pathway [157, 158]. Both subsets of cDCs (CD103^+^ cDC1s and CD11b^+^ cDC2s) are present in the liver of mice and accumulate during NASH [159, 160]. In addition, the number of liver cDC1s is higher in patients with NASH compared with the controls, and the increased number of cDC1s is associated with a greater degree of disease hallmarks of NASH [160]. However, their impact on NASH pathogenesis remains elusive. A recent study showed that the knockout of Atg5 in CD11c^+^ cells promotes the pathogenesis of NAFLD through the production of IL-23 in mice fed an HFD, suggesting a key role of liver DCs in accelerating NASH [161].

## Intestinal immunity in metabolic diseases

In this section, we highlight the roles of intestinal immunity in obesity and diabetes, NASH/NAFLD, and PCOS in terms of mucosal immunity and its effects on the intestinal barrier and other aspects of gut immunity.

### Obesity and diabetes

The intestines display altered immune composition during obesity and function as a crosstalk focus between the intestinal microbiota and intestinal barrier function [162]. In addition to the role of the intestinal physical barrier against gut microbiota, intestinal mucosal immunity is also involved in the regulation of intestinal microbiota. IgA is secreted across the intestinal epithelium in its dimeric form as secretory IgA, which binds to intestinal bacteria and their products to regulate microbiota composition and reduce microbial penetration across the intestinal barrier [163]. Reduced levels of secretory IgA in the stool during DIO in mice lead to dysbiosis, characterized by increased abundance of *Proteobacteria* and decreased abundance of *Clostridia*, accompanied by increased intestinal inflammation and reduced intestinal barrier integrity. In addition, HFD feeding induces a dysfunctional glucose metabolism in IgA-deficient mice [164]. As can be seen, a major consequence of the altered intestinal microbial composition in obesity is increased intestinal permeability, which increases leakage of bacteria or bacterial products, such as lipopolysaccharide (LPS), across the intestinal barrier [165–167]. Bacterial products trigger innate immunity, leading to chronic inflammation and metabolic diseases. LPS infusion for four consecutive weeks recapitulates many of the metabolic abnormalities that occur during ­high-caloric dietary consumption, such as elevated fasting blood sugar and insulin, increased adipose tissue, liver and body weight gain, and increased adipose tissue inflammation [167]. Loss of *Clostridia* and expansion of *Desulfovibrio* were key features associated with obesity, and administration of *Clostridia* rescued obesity by downregulating the expression of genes controlling lipid absorption and reducing adiposity. In addition, it was shown that IgA targeting of *Clostridia* and increased *Desulfovibrio* antagonize the colonization of beneficial *Clostridia*, while T follicular helper cell-dependent events are required to prevent the loss of *Clostridia* and expansion of *Desulfovibrio* [168].

In addition to the regulation of intestinal microbiota toward the metabolic diseases mediated by host intestinal immunity, host immunity can also affect the process of obesity and T2D through direct and indirect regulation of intestinal microbes. Mice fed an HFD show a reduction in the proportion of intestinal Th17 cells. In addition, gut-tropic Th17 cells promote the expansion of intestinal microbiota, including increasing the abundance of *A. muciniphila*, as well as controlling obesity and metabolic disorders via the secretion of IL-17 [169]. *A. muciniphila* has been shown to act as a probiotic that improves abnormal lipid metabolism and IR in mice and individuals with obesity and diabetes [21, 170, 171]. Studies have reported a variety of mechanisms by which *A. muciniphila* affects host metabolism. Administration of *A. muciniphila* reverses HFD-induced metabolic disorders by increasing the intestinal levels of endocannabinoids that control inflammation, improving the intestinal barrier, and promoting the secretion of a gut peptide, such as GLP-1 [20]. This improvement in the intestinal barrier is consistent with the treatment of *A. muciniphila* in mouse models of progeroid and atherosclerosis [172, 173]. In addition, Amuc_1100, a specific protein isolated from the outer membrane of *A. muciniphila*, has been reported to interact with Toll-like receptor 2 (TLR2) to improve the gut barrier, and its administration partly recapitulates the beneficial effects of the bacterium [22]. Recently, studies have shown that by interacting with TLR2, Amuc_1100 promotes the expression of 5-hydroxytryptamine (5-HT) in the intestine, which was negatively correlated with obesity in college students [174, 175]. SFB was identified as a Th17-induced bacteria that promotes the differentiation of Th17 cells by stimulating the secretion of serum amyloid A [176, 177]. Kawano *et al*. showed that HFD feeding eliminates the Th17 cell-inducing microbiota, including SFB, which disrupts Th17 cell-mediated intestinal immunity. Th17 cells were subsequently shown by the authors to protect mice from DIO and metabolic disease by regulating epithelial lipid absorption [13].

### NAFLD and NASH

Like the disruption of the intestinal barrier exacerbated by obesity and diabetes, intestinal microbiota-induced intestinal barrier ­alteration also regulates the pathogenesis of NASH and NAFLD [178]. We have previously mentioned that HFD feeding damages the intestinal barrier in mice [179]. Using a dextran sulfate sodium (DSS)-induced colitis model, a model of intestinal barrier disruption, it was shown that exacerbation of diet-induced NASH in mice follows a breakdown of the intestinal barrier that is accompanied by an elevation of LPS in the serum [180]. A clinical study implied that fecal microbiota transplantation from healthy patients improves abnormal small intestinal permeability in patients with NAFLD, suggesting the important role of gut microbes in regulating the intestinal barrier in NAFLD and NASH [181]. Interestingly, enhanced mitochondrial activity was reported to reshape the intestinal microbiota composition, thereby delaying the progression of NASH, which is associated with a recovery of the intestinal barrier [182]. Importantly, disruption of the gut vascular barrier (GVB) and intestinal epithelial barrier is an early event in NASH pathogenesis, which was shown to be a prerequisite for the development of NASH [183]. By restoring the intestinal barrier, *A. muciniphila* was shown to ameliorate NAFLD [184, 185].

In addition to improving the intestinal barrier, *A. muciniphila* suppresses NASH-associated tumorigenesis through CXCR6^+^ natural killer T cells (NKT) [186]. Although Th17 cells in the liver exacerbate NASH progression, they are decreased in numbers in the small intestine of mice in a methionine choline-deficient (MCD) diet-induced NASH model, suggesting a different function for the cells in this tissue. Indeed, it has been shown that intestinal Th17 cells and IL-17 slow the progression of NAFLD/NASH, which is associated with the restoration of the intestinal barrier that is dependent on the gut microbiota [187]. Disruption of the intestinal barrier is thought to be a risk factor for NASH [179, 180]. As Th17 cell and IL-17 have been reported to improve the intestinal barrier and host MetS, we speculate that intestinal Th17 cells and IL-17 may play a protective role in the progression of NASH and NAFLD [13, 188, 189]. In addition to its role in the gut, Th17 has also been reported to migrate to the liver to aggravate hepatitis [190]. *Candida albicans*-specific Th17 cells migrate from the intestine to the liver where they activate the KCs through IL-17RA and aggravate ­alcohol-associated liver disease [190]. CX3CR1, a marker of NK cells, cytotoxic T lymphocytes, and macrophages, protects mice from excessive hepatic steatosis and inflammation that is accompanied by the regulation of the intestinal barrier. Depletion of intestinal microbiota by antibiotics promotes macrophage polarization in the liver, as well as improving steatohepatitis in mice [191].

### PCOS

Clinical research has shown that zonulin and fatty acid-binding protein 2, two intestinal permeability markers, are not altered in women with PCOS compared with BMI-matched controls [192]. However, Lindheim *et al*. showed that serum diamine oxidase, a marker of intestinal epithelial damage, is significantly higher in patients with PCOS compared with controls [193]. More studies are expected to explore the relationship between PCOS and intestinal barrier.

## Crosstalk between BA metabolism, immune, and intestinal microbiota in different metabolic diseases

**Figure 3:**
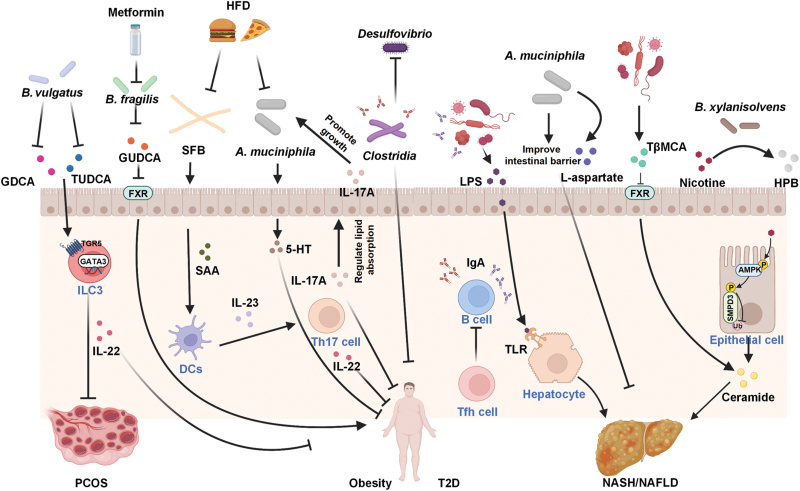
Crosstalk between BA metabolism, immunity, and intestinal microbiota in POCS, obesity, T2D, and NASH/NAFLD.

By exploring the crosstalk between immunity and BA metabolism, we summarize the mechanisms of intestinal microbial regulation of metabolic diseases (Fig. 3 and Table 2).

**Table 2:** Crosstalk between BA metabolism, immunity, and intestinal microbiota in metabolic diseases.

	Subject	Effect and mechanism
T2D and obesity	B cell-derived IgA	Binds to intestinal bacteria and their products [163]. Reduced IgA is associated with decreased *Clostridia* and increased *Proteobacteria*, as well as reduced intestinal barrier integrity and exacerbated dysfunctional glucose metabolism [164].
	Bacteria-derived LPS	Acroases the intestinal barrier and binds to TLR [165–167]. Promotes adipose tissue inflammation and metabolic abnormalities [167].
	*Clostridia* and IgA	*Clostridia* rescues obesity by downregulating the expression of genes controlling lipid absorption and reducing adiposity. IgA targeting beneficial *Clostridia* and increasing of *Desulfovibrio* is prevented by T cells [168].
	Th17 cells	Increase the abundance of *Akkermansia muciniphila* and control obesity and metabolic disorders via secretion of IL-17 [169]. HFD eliminates the Th17 cell-inducing SFB and disrupts the intestinal immune-mediated by Th17 cells, which protect mice from DIO by regulating epithelial lipid absorption [13].
	*A. muciniphila*	Reverses HFD-induced metabolic disorders by increasing the intestinal levels of endocannabinoids that control inflammation, improving the intestinal barrier, and promoting the secretion of gut peptide [20].
	Amuc_1100 of **A.* muciniphila*	Interacts with TLR2, improves the gut barrier, and improves abnormal lipid metabolism and IR in obese and diabetes mice [22]. Promotes the expression of the 5-HT in the intestine by interacting with TLR2 [174].
	*Bacteroides fragilis*	Reverses the metabolic improvement of metformin by decreasing the TUDCA level accompanied by the reduction of BSH activity.
NASH/NAFLD	Bacteria-derived LPS	Exacerbates diet-induced NASH in the DSS model [180].
	*A. muciniphila*	Ameliorates NAFLD by the restoration of intestinal barrier [184, 185]. Suppresses NASH-associated tumorigenesis through expansion of CXCR6^+^ NKT cells [186]. Ameliorates oxidative stress-induced cell apoptosis, increased the levels of l-aspartate in the liver [201].
	*A. muciniphila-*derived l-aspartate	Ameliorates MAFLD in mice [201].
	Five *Faecalibacterium prausnitzii* strains	Improves metabolic indicators and ameliorates NAFLD by the production of SCFAs as well as regulation of gut microbiota and amino acid metabolism [202].
	Intestinal Th17 cells	Decreased in MCD diet-induced NASH model and weakens NAFLD/NASH progress with the restoration of the intestinal barrier depending on the gut microbiota [187].
	Macrophage	Lack of CX3CR1 was associated with NASH, disruption of the intestinal barrier, and microbiota. Depletion of intestinal microbiota promotes macrophage polarization in the liver and improved NASH [191].
	B cell	B-cell deficiency ameliorates the progression of NASH through MyD88 signaling. FMT from NAFLD patients promotes the pathogenesis of NASH mice by increasing the activation and accumulation of intrahepatic B cells [251].
	*A. muciniphila* and quercetin	Ameliorate early obesity and NAFLD through remodeling intestinal microbiota, elevating unconjugated hydrophilic BAs plasma levels, and increasing hepatic expression of BA synthesis and transport genes [203].
	OCA	Protects against GVB disruption and improves the histological features of NASH [183, 204].
	Dietary-derived BAs and FXR agonist GSK2324	GSK2324 controls hepatic lipids via reduced absorption and selective decreases in fatty acid synthesis and BAs prevented decreased lipid absorption in GSK2324-treated mice [205].
	TUDCA	Attenuates hepatic steatosis and IR in mice through reconstitution of gut microbiota and improvement of intestinal barrier [206].
	Gut microbiota and TβMCA	Ablation of gut microbiota alleviates obesity-induced hepatic steatosis dependent on the increasing of TβMCA which inhibits the FXR signaling [207].
	*Bacteroides xylanisolvens*	Reduces intestinal concentrations of nicotine, which activates AMPKα1 phosphorylation, which binds to SMPD3, which mediates ceramide synthesis and exacerbates NASH progression [46].
PCOS	*Bifidobacterium lactis*	Decreases the levels of LH and LH/FSH in nine volunteers as well as increases the levels of SCFAs [211].
	*Lactobacillus*	Improves the PCOS phenotypes and administration of *Lactobacillus* reduces androgen biosynthesis [56].
	*Bacteroides vulgatus*, GDCA and TUDCA	*B. vulgatus* disrupts insulin sensitivity and ovarian function, resulting in a decreased level of TUDCA and infiltration of RORγT^+^ IL-22^+^ ILC3s in siLP. GDCA promotes the production of IL-22 as well as improved IR and ovarian dysfunction in PCOS-like mice by activating the GATA3 signaling pathway [6].

### Obesity and diabetes

*Eggerthella lenta* DSM2243, *Bacteroides fragilis* NCTC9343, and *Ruminococcus gnavus* ATCC29149 convert LCA to 3-oxolithocholic acid and isolithocholic acid (isoLCA) by bacterial hydroxysteroid dehydrogenases [194]. IsoLCA inhibits Th17 cell differentiation by directly binding to the key transcription factor RORγt and increases the differentiation of Treg cells through the production of mitochondrial ROS [195]. Myeloid differentiation ­primary-response gene 88 (MyD88) is the key adaptor for most TLRs, IL-1 receptor (IL-1R), and IL-18 receptor, which have been shown to play a role in obesity and diabetes [196–198]. Hepatocyte-specific deletion of MyD88 predisposes to inflammation, hepatic IR, and glucose intolerance through modulation of BA metabolism and regulation of FXR [199]. These studies suggest that the effect of BA metabolism on metabolic diseases through the regulation of host immunity also needs further study.

Our previous study showed that tempol, a member of a family of nitroxide compounds, reduces the BSH enzymatic activity in the feces by affecting gut microbiota, including decreased abundance of *Lactobacillus caecum*. This intervention increases the level of TβMCA, and thus decreases obesity and improves IR in mice downstream of inhibition of intestinal FXR [4]. In addition, we demonstrated that administration of metformin decreases the abundance of *B. fragilis*, as well as increasing the levels of GUDCA and TUDCA, in the gut, which is accompanied by the inhibition of intestinal FXR signaling. These results may offer at least one mechanism by which metformin improves glucose metabolism. Furthermore, we showed that treatment with *B. fragilis* reverses the metabolic improvement induced by metformin by reducing the TUDCA levels accompanied by a reduction of BSH activity. Finally, we showed that GUDCA is a novel FXR antagonist that shows therapeutic effects on IR and glucose intolerance [200].

### NAFLD and NASH

Recent research has elaborated on the mechanism by which *A. muciniphila* regulates the pathogenesis of NASH. Treatment with *A. muciniphila* increases mitochondrial oxidation and regulates BA metabolism in the gut–liver axis, ameliorating oxidative stress-induced cell apoptosis in the intestine and thus leading to the reshaping of the intestinal microbiota. These metabolic improvements occurred with increased l-aspartate levels in the liver, which was transported from the gut. Meanwhile, administration of l-aspartate displayed beneficial metabolic effects and efficiently ameliorated metabolic dysfunction-associated fatty liver disease (MAFLD) in mice [201]. Five *F. prausnitzii* strains ameliorate NAFLD in mice, which is associated with the regulation of the gut microbiota and metabolic improvement, including enhanced production of SCFAs and regulation of amino acid metabolism [202]. In addition, the gut microbiota also regulates the development of NASH and NAFLD by affecting BA metabolism. Administration of a combination of *A. muciniphila* and quercetin ameliorated early obesity and NAFLD via remodeling of the intestinal microbiota and elevation of plasma levels of unconjugated hydrophilic BAs, as well as increased hepatic expression of BA synthesis and transport genes [203]. The BA analog and FXR agonist, OCA, has been shown to improve the histological features of NASH in a Phase 3 trial, including fibrosis [204]. Pharmacologic intervention with OCA protected against GVB disruption as a preventive and therapeutic agent [183]. Compared with FXR-induced reductions in TAG levels via an FXR-SHP-SREBP1c *de novo* lipogenesis pathway, it has been shown that FXR activation protects against NAFLD via BA-dependent reductions in lipid absorption [205]. In addition, apart from the regulation of FXR, TUDCA administration attenuates HFD-induced hepatic steatosis, inflammatory responses, obesity, and IR in mice by promoting the reconstitution of the intestinal microbiota and improving the intestinal barrier [206].

Our team has long been engaged in studies on the relationship between the intestinal microbiota, BA metabolism, FXR signaling, and the pathogenesis of NAFLD and NASH. In a previous review, we discussed the promotional effects of FXR signaling in NAFLD [10]. We further demonstrated that ablation of the intestinal microbiota alleviates obesity-induced hepatic steatosis in hamsters. This alleviation relied on the upregulation of cytochrome P450 family 7 subfamily B member 1 (CYP7B1) in the alternative BA synthesis pathway, leading to an increase in TβMCA levels that in turn inhibits FXR signaling [207]. Importantly, in a recent study, we elaborated on the mechanism by which the gut bacteria alleviate smoking-related NASH by degrading gut nicotine. Tobacco smoking is positively correlated with NAFLD [208, 209]. Nicotine accumulation in the gut activates AMPKα1 phosphorylation, which is bound to sphingomyelin phosphodiesterase 3 (SMPD3), a key enzyme in the ceramide biosynthesis pathway, and exacerbates NASH progression. Furthermore, we identified the gut bacterium *B. xylanisolvens* as a nicotine degrader, which reduces intestinal nicotine concentrations in nicotine-exposed mice and improves nicotine-exacerbated NAFLD progression [46].

### PCOS

Though the relationship between the alteration of intestinal microbiota and PCOS has been established, only a few studies have reported the possible mechanisms by which the gut microbiome regulates PCOS. Intestinal microbiota-derived SCFA production may be only one of the mechanisms by which intestinal microorganisms regulate PCOS [210]. Zhang *et al*. identified an imbalance in the gut microbiota of patients with PCOS, showing that *Bifidobacterium*, *Blautia*, and *Faecalibacterium* were significantly more abundant in healthy individuals, whereas *Clostridium* and *Parabacteroides* were enriched in the PCOS group. They treated 14 patients with PCOS with the probiotic *Bifidobacterium lactis* V9 and found that this intervention significantly decreased the levels of luteinizing hormone (LH) and the ratio of LH/follicle-stimulating hormone (LH/FSH) in 9 volunteers. The abundance of *B*. *lactis* V9 was positively associated with the levels of SCFAs. In addition, the administration of *B*. *lactis* V9 changed the intestinal microbiota composition of patients with PCOS, including increasing the colonization of *Akkermansia*, *Faecalibacterium*, *Butyricimonas*, and *Bifidobacterium* [211]. *F. prausnitzii* and *Akkermansia* were reported to produce SCFAs and improve inflammation and host metabolism [212, 213]. The study by Zhang *et al*. showed a possible mechanism by which the microbiome affects PCOS. Namely, SCFAs bound to their receptors expressed on enteroendocrine cell membranes directly stimulate the release of gut–brain mediators, such as peptide YY (PYY) and ghrelin. The increased PYY and ghrelin influence the secretion of sex hormones by the hypophysis and hypothalamus through the gut–brain axis, thus exerting an impact on PCOS symptoms. Androgen-induced gut dysbiosis has been reported to disrupt glucolipid metabolism and endocrinal functions in PCOS [214]. A rat study showed that compared with letrozole-induced PCOS rats, the abundance of *Clostridium*, *Lactobacillus*, and *Ruminococcus* was decreased, while *Prevotella* increased compared with the control group. Transplantation of *Lactobacillus* or fecal microbiota from normal rats improved the PCOS phenotypes, and administration of *Lactobacillus* reduced androgen biosynthesis [56]. In addition, the abundance of *Prevotella* was positively associated with androgen levels, especially androstenedione and testosterone.

A study has shown that tempol ameliorates PCOS by modulating the compositions of the gut microbiota and its serum metabolites, including the reduction of serum stachyose and BA levels in PCOS rats [215]. However, the study lacked a mechanism to explain these effects. In our study, we elucidated the mechanism by which the intestinal microbiome regulates PCOS from the perspective of innate immunity and BA metabolism. First, the abundance of *B. vulgatus* was markedly increased in patients with PCOS compared with the healthy controls. In addition, *B. vulgatus* in individuals with PCOS showed an increase in the abundance of BSH genes, both of which lead to a reduction of GDCA and TUDCA in the stool and serum. Administration of *B. vulgatus* disrupted insulin sensitivity and ovarian function, resulting in a decreased level of TUDCA and infiltration of RORγT^+^ IL-22^+^ ILC3s in small intestine lamina propria (siLP). We then identified the preventive effect of IL-22 and GDCA in a prenatal anti-Müllerian hormone-induced PCOS model. By activating the GATA binding protein 3 (GATA3) signaling pathway, GDCA promotes the production of IL-22 dependent on the TGR5 receptor of ILC3s, as well as improving IR and ovarian dysfunction, in PCOS-like mice [6].

## Conclusions and perspective

Mammals have trillions of microbes colonizing their intestines. These mutually beneficial microbes can affect many physiological functions of the host, such as regulating the host’s metabolism, enhancing the intestinal barrier function, and regulating the host’s immune function. In addition to understanding the relevant mechanisms, elucidation of microbial regulation of host immunity and metabolism provides new ideas and targets for the clinical treatment of metabolic diseases. *A. muciniphila* has been reported to improve the metabolism of mice and humans with obesity and diabetes [21, 22]. Although there is no direct clinical evidence that probiotics can treat NASH/NAFLD, several studies have demonstrated the enormous potential of gut microbes in the therapy of NASH. Duan *et al*. designed a bacteriophage to target *Enterococcus faecalis*, a cytolysin-positive bacterium that increases hepatitis, and this intervention attenuated alcoholic liver disease [216].

Regulations of probiotics on diseases by BA metabolism are also noteworthy. VSL#3 (a patented probiotic preparation consisting of eight different strains of probiotics) was reported to improve irritable bowel syndrome in patients probably by increasing the deconjugation and absorption of BAs, which resulted in a reduced bile salt load to the colon [217]. Importantly, VSL#3 demonstrated an improved effect on NASH in mice by improving the intestinal barrier and normalizing the dysregulation of BA synthesis and dysbiosis of intestinal microbiota [218, 219]. In a clinical trial, VSL#3 showed beneficial effects on alanine transaminase (ALT) and BMI in obese children with biopsy-proven NAFLD with the increase of GLP-1 [220]. Besides, *Lactobacillus acidophilus* KLDS1.0901 protected mice against HFD-induced NAFLD by improving liver characteristics through the improvement of intestinal barrier and reduced LPS level [221]. In a randomized controlled trial, combination of *L. acidophilus* and *B. lactis* decreased aspartate transaminase (AST) to platelet ratio index score and AST level in NASH patients.

Many human microbiota studies have confirmed the associations between the gut microbiome and their metabolites, BAs, and the development of metabolic diseases. However, the mechanism by which changes in gut microbiota abundance and/or function regulate the occurrence and development of metabolic diseases has not been fully elucidated. With further study on the function of microbiota, researchers have come to realize that gut microbiome can regulate the metabolic homeostasis by interacting with the host immune system through its metabolites and related receptors. Therefore, in recent years, engineered bacteria have been developed to act on the host metabolism by secreting bacterial immune factors. Hendrikx *et al*. engineered *Lactobacillus reuteri* to produce IL-22 in the intestine to induce expression of regenerating islet-derived 3 gamma (REG3G) and thus reduce ethanol-induced liver disease in mice [222]. In addition, the study of microbial metabolites also provides hints for the clinical treatment of metabolic diseases. As a kind of key endogenous metabolites, BAs have a wide range of effects on various physiological and pathological processes. For example, artificial BA analogs OCA and norUDCA have shown efficacy in clinical trials for NASH [204, 223]. However, OCA may not be approved for the NASH resolution as endpoint was not met. In addition, BAs may have potential off-target effects (such as pruritus, which can be triggered by multiple BAs, and is also a prominent side effect of the major potential clinical BA drug OCA in the field of hepatobiliary diseases) and thus it is difficult to specifically target a disease through modulation of BAs [204, 224]. As a proven drug for the treatment of PSC, UDCA showed controversial efficacy in the clinical therapy of NAFLD [225, 226]. While both UDCA and norUDCA improve NASH, norUDCA, unlike UDCA, offers protection against the development of steatosis and fibrosis, possibly through mechanisms independent of GPBAR1 (TGR5) and FXR [227]. The distinct mechanism of action of norUDCA has shown promising clinical results for NASH [228]. Another potential therapeutic option for NASH is INT-767, a semisynthetic BA that improves histopathological features in NASH mice by activating both FXR and TGR5 [229]. Tropifexor, on the other hand, is a selective, non-BA FXR agonist that has demonstrated high potency in target engagement and efficacy in animal models of NASH [230, 231]. In a Phase 2a/b clinical trial, tropifexor decreased ALT and hepatic fat fraction. However, like FXR agonists, tropifexor can cause pruritus in a dose-dependent manner [232]. In an *in vitro* study, both OCA and INT-767 reversed FFA-induced collagen deposition and metalloproteinase (MMP) reduction in HSCs. Tropifexor, although it reduced collagen deposition, did not show modulation of MMP levels [233]. As can be seen, the therapy strategy of metabolic diseases regarding intestinal microorganisms and microbiota-derived BA metabolites as drugs or targets is promising, but also tortuous. Meanwhile, encouragingly, immune targets for NASH/NAFLD are also undergoing clinical trials and preclinical trials, including an anti-IL-17 antibody and an anti-TNF-α drug (Pentoxyfylline) [130, 234, 235].

As a chronic disease, one of the future research directions of targeting intestinal microbes to treat metabolic disease is editing and modification of bacteria after thorough research on bacterial metabolism and host interactions. In this manner, edited intestinal microbes can be colonized once and work for a long time until they are no longer needed and can be cleaned out using the suicide system [236, 237]. Notably, engineered bacteria are currently safe and effective in the clinical treatment of phenylketonuria [238, 239]. However, *E. coli* Nissle 1917, as an easy-to-manipulate chassis, has a synthetic gene for colibactin which promotes the formation of DNA double-stranded breaks in mammalian DNA [240]. In the future, synthetic biology technology should be considered to target the elimination of harmful genes to ensure the safety of the host.

Historically, researchers have primarily relied on feces and colon contents to assess the composition of gut microbes and gut metabolites. However, it is important to note that pH, nutrient availability, and oxygen partial pressure can vary among regions of the intestine. Consequently, specific intestinal microbes occupying distinct functional and immune niches can be found in specific intestinal regions [241, 242]. Therefore, it became crucial for researchers to develop a method that allows for the collection of intestinal contents from different regions without causing disturbance. In response to this need, Shalon *et al*. developed a capsule device with a built-in collector, enabling the collection of contents from various regions of the intestine based on pH and intestinal retention time. Through their evaluation of this device, they demonstrated that the composition of intestinal microbiota and BA metabolites differed significantly across different intestinal regions [243].

In general, in this review, we discussed the relationship between intestinal microbiota, host immunity, BA metabolism, and metabolic diseases. By expounding on the crosstalk between intestinal microbiota, the immune system, and BA metabolism, we highlighted the mechanisms by which intestinal microbiota contribute to or ameliorate metabolic diseases, as well as providing a new perspective for the understanding of the pathogenesis of metabolic diseases and their clinical treatment.
